# Letter to the editor: Six‐month antibody kinetics and durability in liver transplant recipients after two doses of SARS‐CoV‐2 mRNA vaccination

**DOI:** 10.1002/hep4.2027

**Published:** 2022-07-05

**Authors:** Amy Chang, Alexandra T. Strauss, Jennifer L. Alejo, Teresa P.‐Y. Chiang, Nicole F. Hernandez, Laura B. Zeiser, Brian J. Boyarsky, Robin K. Avery, Aaron A. R. Tobian, Macey L. Levan, Daniel S. Warren, Jacqueline M. Garonzik‐Wang, Allan B. Massie, William A. Werbel, Dorry L. Segev

**Affiliations:** ^1^ Department of Surgery Johns Hopkins University School of Medicine Baltimore Maryland USA; ^2^ Department of Medicine Johns Hopkins University School of Medicine Baltimore Maryland USA; ^3^ Department of Surgery New York University Grossman School of Medicine New York New York USA; ^4^ Department of Population of Health New York University Grossman School of Medicine New York New York USA; ^5^ Department of Surgery University of Wisconsin School of Medicine and Public Health Madison Wisconsin USA

## Abstract

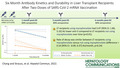

Anti‐spike antibody waning after two doses of severe acute respiratory syndrome coronavirus 2 (SARS‐CoV‐2) messenger RNA (mRNA) vaccination among liver transplant (LT) recipients is anticipated, but the peak and decay kinetics might vary by patient phenotype.^[^ ^1^, ^2^, ^3^ ^]^ This study evaluated 6‐month durability of antibody titers in LT recipients following two‐dose homologous mRNA vaccination, focusing on the impact of antimetabolite use.

Adult LT‐only recipients without reported SARS‐CoV‐2 infection who had antibody titers measured at 1 month and 3–6 months following homologous mRNA vaccine series (D2) were included from a national observational study (IRB00248540).^[^ ^1^ ^]^ Seroconversion was assessed using the Roche Elecsys anti‐receptor‐binding domain or EUROIMMUN anti‐S1 assays.

Peak and longitudinal anti‐S1 trajectories were compared using multilevel mixed‐effects linear regression with a patient‐level random intercept and an interaction between mycophenolate use and time following D2.

Between July 1, 2021, and October 26, 2021, a total of 161 participants received BNT162b2 (47%) or mRNA‐1273 (53%) vaccination. Median (interquartile range [IQR]) age was 60 (46–67) years, and most were female (56%). Median (IQR) years since transplant was 6 (3–16). Peri‐vaccination immunosuppression regimens included calcineurin inhibitor (87%), antimetabolites (38%), corticosteroids (22%), and mammalian target of rapamycin inhibitor (16%); 20% or 7% received dual (calcineurin inhibitors and steroids) or triple immunosuppression (dual immunosuppression and antimetabolites), respectively.

Overall, 136 of 161 (84%) tested seropositive at a median (IQR) of 30 (28–32) days after D2. Of those with available paired titers, 133 of 149 (89%) were seropositive at 3 months, and 49 of 58 (84%) were seropositive at 6 months following D2. Of the 7 seronegative persons with paired titers, 4 (57%) seroconverted to low‐level positive titer by 6 months.^[^ ^4^ ^]^

Participants taking mycophenolate were more likely to be seronegative at 1 month (22 of 53 [42%] vs. 3 of 108 [2%]; *p* < 0.001) and 3 months (13 of 50 [26%] vs. 3 of 99 [3%]; *p* < 0.001) following D2. Additionally, participants taking mycophenolate had lower peak antibody levels (−3.47 AU [−5.10, −1.80]; *p* < 0.01), albeit similar rate of decay by 6 months following D2 (difference of +0.14 AU/month [−0.09, 0.37]; *p* = 0.24) (Figure 1).

**FIGURE 1:**
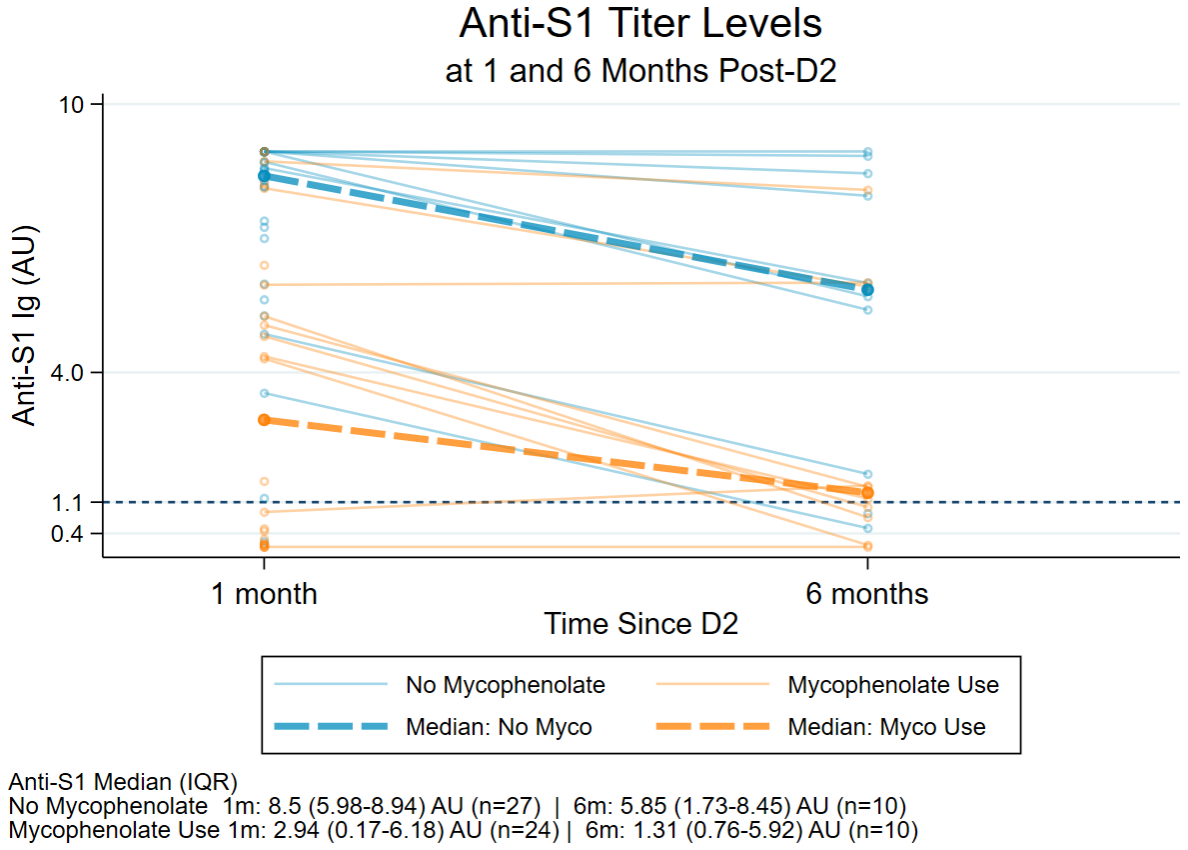
Anti‐S1 antibody level at 1 month and 6 months following a second messenger RNA (mRNA) severe acute respiratory syndrome coronavirus 2 (SARS‐CoV‐2) vaccine (post‐D2), categorized by mycophenolate use in liver transplant recipients. Abbreviation: IQR, interquartile range.

Seroconversion following two‐dose mRNA vaccination in LT recipients was high and persisted for up to 6 months, although with steady decline. Mycophenolate use was negatively associated with sero‐response, yet did not impact decay kinetics, implying a mechanism of interference with initial vaccine response rather than accelerated waning. This study was limited by absence of serological testing for viral exposure and an assessment of real‐world vaccine efficacy.

## CONFLICT OF INTEREST

D. L. Segev consults for and is on the speakers bureau for Sanofi, Novartis, CLS Behring, Jazz Pharmaceuticals, Veloxis, Mallinckrodt, Thermo Fisher Scientific, Regeneron, and AstraZeneca. R. K. Avery receives study/grant support from Aicuris, Astellas, Chimerix, Merck, Oxford Immunotec, Qiagen, Regeneron, Takeda/Shire, and Vir/GSK, and is an associate reviewer for *Transplantation*. M. L. Levan is the social media editor for *Transplantation* and is a consultant for Takeda/Shire and Patients Like Me.

## FUNDING INFORMATION

This research was made possible by the generous support of the Ben‐Dov family and the Trokhan Patterson family. This work was supported by the National Institute of Diabetes and Digestive and Kidney Diseases (T32DK007732, T32DK007713, F32DK124941, K01DK114388–03, K01DK101677, and K23DK115908); the Pearl M. Stetler Research Fund from the Johns Hopkins School of Medicine; and the National Institute of Allergy and Infectious Disease (K24AI144954, K23AI157893, and U01AI138897). The analyses described here are the responsibility of the authors alone and do not necessarily reflect the views or policies of the Department of Health and Human Services, nor does mention of trade names, commercial products, or organizations imply endorsement by the US government.
